# Exploring the Associations between Single-Child Status and Childhood High Blood Pressure and the Mediation Effect of Lifestyle Behaviors

**DOI:** 10.3390/nu14030500

**Published:** 2022-01-24

**Authors:** Rui Deng, Ke Lou, Siliang Zhou, Xingxiu Li, Zhiyong Zou, Jun Ma, Bin Dong, Jie Hu

**Affiliations:** 1 Institute of Child and Adolescent Health, School of Public Health, Peking University Health Science Center, Beijing 100191, China; dengrui@stu.pku.edu.cn (R.D.); louke@pku.edu.cn (K.L.); slzhou@pku.edu.cn (S.Z.); 1610306239@pku.edu.cn (X.L.); harveyzou2002@bjmu.edu.cn (Z.Z.); majunt@bjmu.edu.cn (J.M.); 2 Menzies Health Institute, Griffith University, Nathan, QLS 4111, Australia; jie.hu@griffith.edu.au

**Keywords:** children, blood pressure, single-child status, lifestyle behaviors

## Abstract

Background: This study aimed to assess the association between single-child status and childhood high blood pressure (HBP) and to explore the role of lifestyle behaviors in this relationship. Methods: This study used data from a cross-sectional survey of 50,691 children aged 7~18 years in China. Linear and logistic regression models were used to assess the relationship between single-child status and HBP, and interactions between single-child status and lifestyle behaviors were also evaluated. Mediation analysis was conducted to detect the mediation effect of lifestyle behaviors. Results: Of the participants enrolled, 67.2% were single children and 49.4% were girls. Non-single children were associated with a greater risk of HBP, especially in girls (OR = 1.11, 95%CI: 1.03~1.19). Meat consumption and sedentary behavior mediated 58.9% of the association between single-child status and HBP (*p* < 0.01). When stratified by sleeping duration, non-single girls of insufficient sleep and hypersomnia showed a higher risk of HBP (*p* < 0.05) than single-child peers, but not in those with adequate sleep. Conclusion: Findings suggest that non-single children had an increased risk of HBP, and keeping healthy lifestyle behaviors could help to mitigate the adverse impact in non-single children.

## 1. Introduction

Hypertension is the leading risk for global disability-adjusted life-years (DALYs), accounting for 9.2% of DALYs for men and 7.8% of DALYs for women in 2015 [1]. A Chinese national survey [2] conducted from 2012 to 2015 showed that an estimated 244.5 million adults had hypertension. Evidence has suggested that childhood high blood pressure (HBP) could track to adulthood [3] and increase the risk of cardiovascular disease events in adulthood [4]. A meta-analysis [5] showed that the pooled prevalence of elevated BP among Chinese children and adolescents was 9.8% (95%CI: 7.9~11.9). Thus, preventing hypertension during childhood and identifying children and adolescents with an increased risk of HBP is of far-reaching public health significance.

Currently, countries [6] are struggling with population aging, and various policies have been proposed. In October 2015, China abolished the one-child policy, which lasted for 35 years, and subsequently implemented the universal two-child policy [7]. Soon after, in May 2021, the Chinese government further announced the policy that one couple is allowed to have three children. These new fertility policies may contribute to a large increase in non-single-child families in the future. Thus far, many research studies have investigated the influence of single-child status on health issues, including obesity [8,9,10], poor vision [11], anxiety and depression [12]. However, cardio-metabolic disorders and sing-child status are seldom studied.

Considering the progressing demographic transition in China, we wondered whether the rising proportion of non-single children would change the risk of HBP in the Chinese population, from both an immediate and long-term perspective. Furthermore, previous studies have suggested that lifestyle behaviors might contribute to the differences in body mass index (BMI) between single and non-single children [13]. It is unclear whether lifestyle behaviors could also mediate or modify the relationship between single-child status and HBP.

Using data collected from nationwide Chinese children and adolescents, this study aimed to examine the association of single-child status with childhood HBP and to further investigate the role of lifestyle behaviors in the relationship between single-child status and risk of HBP.

## 2. Materials and Methods

### 2.1. Study Design and Participants

This study used data from the baseline cross-sectional survey of a national school program from seven provinces or cities of China (Guangdong, Shanghai, Hunan, Ningxia, Chongqing, Liaoning, and Tianjin) in 2013. A detailed description of the study design is reported elsewhere [14]. Briefly, using the multistage cluster random sampling method, a total of 93 primary and secondary schools were randomly selected, and 53,532 children and adolescents were involved in the present study. Exclusion criteria included: (a) participants aged out of 7~18 years old (*n* = 250); (b) participants who were not a singleton birth (*n* = 1577); (c) participants with a missing value for blood pressure (*n* = 1014). Thus, a total of 50,691 participants (94.7%) were involved in analyses. The study has been approved by the Ethics Committee of Peking University (No. IRB0000105213034). All participating students and their parents have signed the informed consent.

### 2.2. Anthropometric Measurements

All participants underwent anthropometric and clinical measurements by qualified medical physicians from medical establishments in schools. Height was measured two times using a portable stadiometer (model TZG, China) to the nearest 0.1 cm without shoes. Weight was measured two times using a lever-type weight scale (model RGT-140, China) to the nearest 0.1 kg in light clothing and without shoes. The average of two repeated measures was calculated for height and weight. BMI was calculated as weight in kilograms divided by the square of height in meters (kg/m^2^) and then converted into age- and sex-specific BMI z scores according to the World Health Organization (WHO) growth references [15]. BP was obtained by Mercury sphygmomanometer (model XJ11D, Shanghai Medical Instruments Co, Ltd, Shanghai, China) and stethophone (model TZ-1, Shanghai Medical Instruments Co, Ltd, Shanghai, China) from the right arm using the appropriate cuffs. Prior to the first reading, children were asked to sit quietly for at least 5 min. Systolic blood pressure (SBP) was determined by the onset of the first Korotkoff sound, and diastolic blood pressure (DBP) was determined by the fifth Korotkoff sound. BP was measured twice with a 5 min gap between replicates, and the averages of two readings for SBP and DBP were calculated for each participant.

### 2.3. Questionnaires Data Collection

Questionnaires were designed for students and their parents, separately. Students of grade four or older (≥10 years) finished the student questionnaire independently in a classroom, while children of grade 1~3 (7~9 years) answered the questionnaire with the help of parents at home. Child self-reported lifestyle behaviors were obtained by the student questionnaire, while obstetric information (e.g., gestational age, delivery mode and birth weight) were obtained from the parental questionnaire. Moreover, information on family history of hypertension was also obtained from the parental questionnaire, and children were considered as having a family history of hypertension when either father or mother self-reported that he/she had been diagnosed with hypertension by medical physicians. Both father’s and mother’s education degrees were collected, and the higher one was defined as the parental highest education degree.

### 2.4. Definitions

Single-child status was defined using the question “Is your child the only child?” in the parental questionnaire, and children were assigned to “single children” or “non-single children” groups accordingly. As suggested by the American Academy of Pediatrics [16], high blood pressure (HBP) was defined if child blood pressure was ≥90th percentile or 120/80 mmHg. Lifestyle behaviors, including diet behaviors, physical activity, sedentary behavior and sleeping duration, were investigated in the current study. Diet behaviors were identified as frequencies (days per week) of consuming meat, fruit, vegetables and beverages during the past 7 days. Based on child self-reported frequency (days) and duration (minutes per day) of moderate and vigorous-intensity activity over the past 7 days, physical activity was defined with reference to the International Physical Activity Questionnaire [17]. Sedentary behavior (h) referred to the total time spent sitting or lying down at school or home, including watching television, playing computer games and doing homework. Sleeping duration per day was recorded, and students were divided into three groups (enough sleep (≥9 h), insufficient sleep (7~8 h) and hypersomnia (<7 h)) according to the recommendation of National Sleep Foundations [18].

### 2.5. Statistical Analysis

Continuous variables were reported as mean ± standard deviation, and categorical variables were reported as frequency (percentage). Differences between single children and non-single children were tested by *t* test for continuous variables and χ^2^ test for categorical variables. Linear regression models were used to evaluate the association between single-child status and BP z score, while logistic regression models were used to test the relationship between single-child status and risk of HBP by sex. To explore the potential factors mediating the association of single-child status with HBP, a mediation analysis proposed by Karlson and colleagues, was conducted [19]. This method decomposed the total effect of a variable into direct effect and indirect effect and calculated the percentage of the main association explained by the mediators. Moreover, the interaction terms of single-child status and each lifestyle behavioral factor, such as interaction between single-child status and frequency of meat consumption, were also tested. All analyses were performed with R studio 1.2.5033 and Stata software version 14 (StataCorp, College Station, TX, USA). A two-sided *p* < 0.05 was considered statistically significant.

## 3. Results

Characteristics of participants are shown in Table 1 by sex and single-child status. Among 50,691 participants, 67.2% were single children and 49.4% were girls. Non-single children were associated with higher z scores of BP, while single children were related to a greater BMI z score in both boys and girls (*p* < 0.05). A significant difference in HBP prevalence was observed in single and non-single girls. In both sexes, single children more frequently ate meat, fruit and vegetables, and they spent more time sitting, lying, and sleeping, compared to those children with siblings.

The associations between single-child status and BP levels were assessed by sex, and non-single children generally had higher BP levels compared with single children (Table 2). When stratified by sex, similar relationships were detected in girls after adjusting for confounders (for SBP: β = 0.073, 95%CI: 0.043~0.102, *p* < 0.001; for DBP: β = 0.062, 95% CI: 0.032~0.091, *p* < 0.001; for HBP: OR = 1.11, 95% CI: 1.03~1.19, *p* < 0.05), but not in boys.

Mediation analysis was conducted to identify the mediation effects of lifestyle behaviors on the relationship between single-child status and BP level. In girls (Table 3), frequency of meat consumption mediated about one-third of the association between single-child status and SBP z score (mediation effect = 0.017, 95% CI: 0.013~0.021, *p* < 0.001, mediation ratio = 30.31%) and DBP z score (mediation effect = 0.016, 95% CI: 0.012~0.020, *p* < 0.001, mediation ratio = 31.40%). The corresponding mediation effect for sedentary behavior was 0.002 (*p* < 0.01) with the mediation proportion of 4.02% for SBP and 4.18% for DBP. When HBP was the outcome variable, the mediation proportions of consuming meat and sedentary behavior were 46.98% and 11.87%, with a mediation effect of 0.022 (95% CI: 0.015~0.028, *p* < 0.001) and 0.004 (95%CI: 0.001~0.007, *p* < 0.01), respectively. Similar patterns were also found in boys, especially the mediation effect of consuming meat (Supplementary Material Table S3).

When interaction terms of single-child status and different lifestyle behaviors were analyzed, a significant interaction effect of sleeping duration and single-child status was observed (*p* values < 0.05) (for girls: Supplementary Materials Tables S1 and S2; for boys: Supplementary Materials Tables S4 and S5). Among various sleeping duration groups (Table 4), non-single girls of insufficient sleep had higher BP z scores (for SBP: β = 0.110, 95% CI: 0.072~0.151, *p* < 0.001; for DBP: β = 0.098, 95% CI: 0.058~0.137, *p* < 0.001) than their single peers, with the risk of HBP raised by 15% (OR = 1.15, 95%CI: 1.04~1.27, *p* < 0.05). Similar patterns were also observed in children with hypersomnia, in which SBP z scores and risk of HBP in non-single girls increased by 0.220 (95%CI: 0.019~0.420, *p* < 0.05) and 88% (OR = 1.88, 95% CI: 1.08~3.32, *p* < 0.05), respectively. However, in girls with adequate sleep, the BP levels were similar regardless of single-child status. The above analyses were repeated in boys, and similar patterns were observed, although the associations were mostly not statistically significant.

## 4. Discussion

In the present study, non-single girls were positively associated with HBP in those with insufficient sleeping duration or hypersomnia, but not in those with adequate sleep. These associations were independent of BMI. Furthermore, lifestyle behaviors, such as meat consumption and sedentary behavior, mediated 34.3%~58.9% of the association between single-child status and BP levels. These findings suggested that adherence to a healthy lifestyle could relieve the adverse impact on BP in non-single children.

Our findings are contrary to some previous research. Kelishadi and colleagues [20] found that single Iranian children had an increased risk of SBP (OR = 1.58; 95% CI: 1.17~2.14). Considering that single children were all firstborns, studies [21,22] found that firstborns had higher blood pressure compared with later-born children. However, these studies did not take child BMI into account. It has been established that single children were associated with higher risk of obesity [23,24,25], which was also supported by our study. Besides, BMI was also strongly associated with HBP [26,27]. In the present study, BMI was identified as a confounder, rather than a mediator, in the associations between single-child status and BP, because BMI did not show its mediation effect in the mediation analysis. After controlling for BMI, we concluded that non-single children had a greater risk of HBP than single peers. In previous studies, results of elevated BP levels in single children also disappeared after adjusting for BMI [20] and weight status [22].

In this study, lifestyle behaviors were investigated, with meat consumption and sedentary behavior identified as mediators. Multiple studies have shed light on dietary behaviors (such as meat consumption and eating outside) and sedentary time as the mechanism underlying the association between single-child status and their health outcomes [9,20,28,29]. A systematic literature review [30] showed that peers or siblings often had a negative influence on children’s healthy eating behavior; for example, children may increase consumption of energy-dense foods such as meat because their siblings may tease them about eating fruits and vegetables [31]. Meanwhile, studies have showed that higher meat consumption is related to greater risk of HBP [32,33]. For example, a cohort study [34] found that participants who consumed ≥5 servings of red meat had a 17% higher rate of hypertension compared with those who consumed <1 serving/wk. Although these results could support our findings, the mechanism remains highly speculative, and more studies are needed to provide further evidence.

Our study additionally found that the adverse effect related to single-child status could be modified by improving lifestyle behaviors, such as sufficient sleep. Quist and colleagues reviewed [35] the effect of sleeping on childhood health outcomes and concluded that inadequate sleep was associated with HBP. Another study conducted [36] on American children showed that both short and long sleeping duration was associated with an elevated risk of hypertension, with a stronger association for the short sleeping duration. In our study, we demonstrated that non-single children with adequate sleep were not related to a greater HBP risk, while those of both short or long sleeping duration showed a raised HBP risk. These findings suggested that interventions of a modifiable lifestyle, such as sleeping, could be helpful in relieving the risk of HBP in non-single children.

Furthermore, we found that the association between single-child status and the risk of HBP was only statistically significant among girls. Similarly, a study [37] conducted within the Pelotas 2004 cohort also observed lower SBP and DBP in first-born females, and another study [38] involving over 1 million Swedish men found no birth order differences in BP. In our study, the possible explanation for the gender differences among the Chinese might be partly attributed to the Chinese traditional preference for sons [39]. Chinese boys may have less discrepancy in lifestyle behaviors with their brothers because they equally enjoy the family’s attention and resources, which were more than that for girls [40,41]. As a result, there are limited differences in BP between single and non-single Chinese boys.

Several limitations should be taken into consideration when interpreting the findings. First, causality is difficult to be inferred from this cross-sectional study. Second, some factors, such as maternal and paternal BMI and parent–child interaction, were not investigated. In addition, lifestyle behaviors were collected from self-reported questionnaires, and instrumental measurements are needed to obtain more accurate and objective data, such as ActiGraph GT3X accelerometers. Furthermore, BP measurements were taken in one visit for each individual, which may overestimate the prevalence of HBP. Finally, as this study was conducted in the Chinese population, studies in other countries are warranted to clarify the generalizability of our findings.

## 5. Conclusions

To summarize, this study found that non-single children were associated with a greater risk of HBP than their single-child peers, especially in girls and those with short or prolonged sleeping duration. This association was potentially mediated by lifestyle behaviors, such as meat consumption and sedentary behavior. These findings suggested that keeping a healthy lifestyle could mitigate the disadvantageous impact on BP profile among non-single children and adolescents. More studies are desired to clarify the mechanisms underlying the observations that this study presented.

## Figures and Tables

**Table 1:** Characteristics of participants involved according to single-child status and by sex (*n* = 50,691).

	Boys			Girls		
	Single Children	Non-Single Children	*p* Value	Single Children	Non-Single Children	*p* Value
Number of children	18,397 (71.7)	7264 (28.3)		15,691 (62.7)	9339 (37.3)	
Age, years	10.59 ± 3.2	10.62 ± 3.2	<0.001 ***	10.65 ± 3.27	11.22 ± 3.32	<0.001 ***
Height, cm	146.7 ± 18.3	145.3 ± 18.1	<0.001 ***	144.1 ± 15.5	144.9 ± 15.1	<0.001 ***
Weight, kg	42.7 ± 17.3	40.4 ± 15.7	<0.001 ***	39.2 ± 14.1	39.7 ± 13.5	<0.001 ***
BMI z score	0.48 ± 1.4	0.25 ± 1.3	<0.001 ***	0.07 ± 1.2	−0.04 ± 1.1	<0.001 ***
SBP z score	0.07 ± 1.0	0.11 ± 1.0	0.002 **	−0.10 ± 1.0	0.05 ± 1.0	<0.001 ***
DBP z score	0.04 ± 1.0	0.09 ± 1.0	<0.001 ***	−0.11 ± 1.0	0.01 ± 1.0	<0.001 ***
HBP	5647 (30.7)	2237 (30.8)	0.887	3247 (20.7)	2223 (23.8)	<0.001 ***
Breastfeeding	15,138 (83.5)	6273 (87.7)	<0.001 ***	12,878 (82.9)	8187 (89.1)	<0.001 ***
Birth weight, g	3359.9 ± 497.8	3406.7 ± 533.1	<0.001 ***	3273.7 ± 472.8	3306.3 ± 499.8	<0.001 ***
Caesarean section	8814 (48.6)	2104 (29.5)	<0.001 ***	7410 (47.8)	2416 (26.4)	<0.001 ***
Gestational age, week	39.7 ± 1.2	39.9 ± 1.1	<0.001 ***	39.8 ± 1.21	39.9 ± 1.06	<0.001 ***
Meat consumption per week			<0.001 ***			<0.001 ***
0~1 day(s)	1127 (6.4)	630 (9.0)		1105 (7.3)	1019 (11.3)	
2~3 days	3164 (17.9)	1608 (23.1)		3151 (20.9)	2648 (29.4)	
4~5 days	3013 (17.1)	1340 (19.2)		2692 (17.8)	1749 (19.4)	
6~7 days	10,358 (58.6)	3387 (48.6)		8157 (54.0)	3598 (39.9)	
Fruits consumption per week			<0.001 ***			<0.001 ***
0~1 days	1362 (7.7)	633 (9.1)		703 (4.7)	532 (5.9)	
2~3 days	3674 (20.8)	1753 (25.3)		2612 (17.3)	1927 (21.4)	
4~5 days	4232 (24.0)	1830 (26.4)		3658 (24.2)	2417 (26.9)	
6~7 days	8373 (47.5)	2725 (39.3)		8119 (53.8)	4124 (45.8)	
Vegetable consumption per week			<0.001 ***			<0.001 ***
0~1 day(s)	775 (4.4)	360 (5.2)		563 (3.7)	391 (4.3)	
2~3 days	1408 (8.0)	648 (9.3)		1029 (6.8)	857 (9.5)	
4~5 days	1989 (11.3)	938 (13.5)		1522 (10.1)	1132 (12.6)	
6~7 days	13,496 (76.4)	5021 (72.1)		11,990 (79.4)	6632 (73.6)	
Beverage consumption per week			0.502			0.071
0 day	5237 (29.8)	2035 (29.3)		5477 (36.4)	3148 (35.0)	
1~2 days	7796 (44.4)	3137 (45.2)		6724 (22.8)	4111 (45.7)	
3 days and more	4530 (25.8)	1766 (25.5)		2835 (18.9)	1745 (19.4)	
Physical activity			0.051			<0.001 ***
Low intensity	2533 (15.9)	932 (15.2)		2457 (18.0)	1477 (18.6)	
Median intensity	4970 (31.1)	1847 (30.0)		5354 (39.1)	2874 (36.2)	
High intensity	8454 (53.0)	3369 (54.8)		5870 (42.9)	3580 (45.1)	
Sedentary behavior, h	5.54 ± 3.6	5.25 ± 3.7	<0.001 ***	5.90 ± 3.7	5.64 ± 3.7	<0.001 ***
Sleeping duration			0.020 *			<0.001 ***
Adequate sleep	5908 (35.6)	2391 (36.7)		4931 (34.2)	3006 (35.2)	
Insufficient sleep	10,275 (61.9)	3938 (60.4)		9264 (64.2)	5324 (62.4)	
Hypersomnia	403 (2.4)	191 (2.9)		242 (1.7)	203 (2.4)	
Having a family history of hypertension	1157 (6.6)	495 (7.2)	0.091	1063 (7.0)	662 (7.4)	0.278
Parental highest education degree			<0.001 ***			<0.001 ***
Illiteracy or elementary school	342 (1.9)	496 (7.1)		246 (1.6)	573 (6.2)	
Junior high school	4639 (26.0)	3542 (51.0)		3108 (20.4)	4662 (51.9)	
Senior high school	5248 (29.4)	1928 (27.8)		4454 (29.2)	2466 (27.4)	
Technical secondary school/ Junior college	3596 (20.1)	586 (8.4)		3492 (22.9)	771 (8.6)	
Undergraduate or above	4033 (22.6)	394 (5.7)		3930 (25.8)	516 (5.7)	

Note: Continuous variables were expressed by mean values ± standard deviations, and categorical variables were expressed by frequency value (percentages, %). BMI, body mass index; HBP, high blood pressure; SBP, systolic blood pressure; DBP, diastolic blood pressure. * *p* < 0.05, ** *p* < 0.01, *** *p* < 0.001.

**Table 2:** Associations between single-child status and childhood BP.

	Model 1		Model 2	
	β/OR (95% CI)	*p* Value	β/OR (95% CI)	*p* Value
SBP z score				
Total				
Single children	0 (ref)		0 (ref)	
Non-single children	0.123 (0.084~0.121)	<0.001 ***	0.037 (0.016~0.058)	<0.001 ***
Boys				
Single children	0 (ref)		0 (ref)	
Non-single children	0.043 (0.016~0.070)	0.002 **	0.005 (−0.025~0.035)	0.735
Girls				
Single children	0 (ref)		0 (ref)	
Non-single children	0.161 (0.135~0.186)	<0.001 ***	0.073 (0.043~0.102)	<0.001 ***
DBP z score				
Total				
Single children	0 (ref)		0 (ref)	
Non-single children	0.095 (0.076~0.113)	<0.001 ***	0.035 (0.014~0.056)	0.001 **
Boys				
Single children	0 (ref)		0 (ref)	
Non-single children	0.055 (0.029~0.082)	<0.001 ***	0.013 (−0.017~0.043)	0.404
Girls				
Single children	0 (ref)		0 (ref)	
Non-single children	0.137 (0.111~0.163)	<0.001 ***	0.062 (0.032~0.091)	<0.001 ***
HBP				
Total				
Single children	1 (ref)		1 (ref)	
Non-single children	1.08 (1.03~1.12)	0.001 **	1.00 (0.95~1.05)	0.884
Boys				
Single children	1 (ref)		1 (ref)	
Non-single children	1.00 (0.94~1.06)	0.956	1.00 (0.93~1.07)	0.991
Girls				
Single children	1 (ref)		1 (ref)	
Non-single children	1.20 (1.13~1.27)	<0.001 ***	1.11 (1.03~1.19)	0.008 **

Model 1 adjusted for age and sex if the variable was not the grouping variable. Model 2 adjusted for age, birth weight, gestational age, breastfeeding, delivery mode, family history of hypertension, parental highest education degree, BMI z score if the variable was not the grouping variable. BP, blood pressure; SBP, systolic blood pressure; DBP, diastolic blood pressure; HBP, high blood pressure. ** *p* < 0.01, *** *p* < 0.001.

**Table 3:** Mediation of lifestyle behaviors on the association between single-child status and BP in girls (*n* = 25,030).

Mediator	*n*	Outcome	Total Association		Direct Association		Indirect Association		Mediation Proportion
			β (95% CI)	*p*	β (95% CI)	*p*	β (95% CI)	*p*	
Meat consumption per week	20,053	SBP z score	0.059 (0.025~0.087)	<0.001 ***	0.039 (0.008~0.070)	0.014 **	0.017 (0.013~0.021)	<0.001 ***	30.31%
	20,054	DBP z score	0.050 (0.003~0.065)	0.002 **	0.034 (0.003~0.065)	0.032 *	0.016 (0.012~0.020)	<0.001 ***	31.40%
	29,957	HBP	0.046 (−0.030~0.121)	0.234	0.024 (−0.051~0.100)	0.529	0.022 (0.015~0.028)	<0.001 ***	46.98%
Fruits consumption per week	20,049	SBP z score	0.056 (0.024~0.087)	<0.001 ***	0.058 (0.027~0.089)	<0.001 ***	—	—	—
	20,050	DBP z score	0.050 (0.019~0.081)	0.002 **	0.053 (0.021~0.083)	0.001 **	—	—	—
	20,053	HBP	0.045 (−0.031~0.120)	0.247	0.050 (−0.026~0.125)	0.195	—	—	—
Vegetable consumption per week	20,053	SBP z score	0.057 (0.026~0.088)	<0.001 ***	0.057 (0.026~0.088)	<0.001 ***	—	—	—
	20,054	DBP z score	0.051 (0.020~0.082)	0.001 **	0.052 (0.021~0.083)	0.001 **	—	—	—
	20,057	HBP	0.049 (−0.030~0.125)	0.200	0.052 (−0.023~0.127)	0.176	—	—	—
Beverage consumption per week	19,980	SBP z score	0.056 (0.024~0.087)	<0.001 ***	0.055 (0.024~0.087)	0.001 **	0.000 (−0.000~0.001)	0.304	—
	19,981	DBP z score	0.049 (0.018~0.080)	0.002 **	0.049 (0.017~0.080)	0.002 **	0.000 (−0.001~0.001)	0.364	—
	19,984	HBP	0.046 (−0.029~0.122)	0.231	0.045 (−0.030~0.121)	0.239	0.001 (−0.001~0.002)	0.411	—
Physical activity	18,181	SBP z score	0.065 (0.032~0.098)	<0.001 ***	0.065 (0.032~0.098)	<0.001 ***	—	—	—
	18,182	DBP z score	0.059 (0.026~0.092)	<0.001 ***	0.059 (0.026~0.092)	<0.001 ***	0.000 (−0.000~0.000)	0.895	—
	18,185	HBP	0.055 (−0.024~0.134)	0.173	0.055 (−0.024~0.134)	0.174	0.000 (−0.002~0.002)	0.962	—
Sedentary behavior	18,256	SBP z score	0.052 (0.020~0.085)	0.002 **	0.050 (0.017~0.083)	0.003 **	0.002 (0.001~0.004)	0.004 **	4.02%
	18,257	DBP z score	0.044 (0.011~0.076)	0.009 **	0.042 (0.009~0.075)	0.012 *	0.002 (0.001~0.003)	0.007 **	4.18%
	18,260	HBP	0.036 (−0.043~0.115)	0.373	0.032 (−0.047~0.110)	0.432	0.004 (0.001~0.007)	0.008 **	11.87%
Sleeping duration	19,211	SBP z score	0.054 (0.220~0.086)	0.001 **	0.054 (0.022~0.086)	0.001 **	—	—	—
	19,212	DBP z score	0.048 (0.016~0.080)	0.003 **	0.048 (0.016~0.080)	0.003 **	0.000 (−0.000~0.000)	0.902	—
	19,215	HBP	0.046 (−0.031~0.123)	0.243	0.046 (−0.031~0.123)	0.243	0.000 (−0.000~0.000)	0.978	—

Note: The independent variable in the model was single-child status and the reference group was “single children”. Adjusted for age, birth weight, gestational age, breastfeeding, delivery mode, family history of hypertension, parental highest education degree, BMI z score. “—” represents the negative value of indirect association. BP, blood pressure; SBP, systolic blood pressure; DBP, diastolic blood pressure; HBP, high blood pressure. * *p* < 0.05, ** *p* < 0.01, *** *p* < 0.001.

**Table 4:** Associations between single-child status with childhood BP stratified by sex and sleeping duration.

Sleeping Duration	SBP z Score		DBP z Score		HBP	
	β (95% CI)	*p* Value	β (95% CI)	*p* Value	OR (95% CI)	*p* Value
Girls						
Adequate sleep						
Single children	0 (ref)		0 (ref)		1 (ref)	
Non-single children	−0.007 (−0.060~0.046)	0.794	−0.003 (−0.057~0.052)	0.927	0.99 (0.86~1.13)	0.860
Insufficient sleep						
Single children	0 (ref)		0 (ref)		1 (ref)	
Non-single children	0.110 (0.072~0.151)	<0.001 ***	0.098 (0.058~0.137)	<0.001 ***	1.15 (1.04~1.27)	0.006 **
Hypersomnia						
Single children	0 (ref)		0 (ref)		1 (ref)	
Non-single children	0.220 (0.019~0.420)	0.032 *	0.149 (−0.071~0.369)	0.184	1.88 (1.08~3.32)	0.026 *
Boys						
Adequate sleep						
Single children	0 (ref)		0 (ref)		1 (ref)	
Non-single children	−0.025 (−0.078~0.028)	0.351	−0.049 (−0.103~0.005)	0.074	0.88 (0.78~1.00)	0.058
Insufficient sleep						
Single children	0 (ref)		0 (ref)		1 (ref)	
Non-single children	0.020 (−0.021~0.061)	0.336	0.056 (0.015~0.098)	0.008 **	1.06 (0.96~1.17)	0.236
Hypersomnia						
Single children	0 (ref)		0 (ref)		1 (ref)	
Non-single children	0.031 (−0.163~0.225)	0.755	−0.070 (−0.278~0.137)	0.505	1.12 (0.70~1.76)	0.639

Adjusted for age, birth weight, gestational age, breastfeeding, delivery mode, family history of hypertension, parental highest education degree, BMI z score. BP, blood pressure; SBP, systolic blood pressure; DBP, diastolic blood pressure; HBP, high blood pressure. * *p* < 0.05, ** *p* < 0.01, *** *p* < 0.001.

## Data Availability

The data supporting the conclusions of this article will be made available from the corresponding author upon request.

## Supplementary Materials

The following supporting information can be downloaded at: https://www.mdpi.com/article/10.3390/nu14030500/s1, Table S1: Moderation of lifestyle behaviors on the association between single-child status and BP z score in girls (*n* = 25,030), Table S2: Moderation of lifestyle behaviors on the association between single-child status and HBP in girls (*n* = 25,030), Table S3: Mediation of lifestyle behaviors on the association between single-child status and BP in boys (*n* = 25,661), Table S4: Moderation of lifestyle behaviors on the association between single-child status and BP z score in boys (*n* = 25,661), Table S5: Moderation of lifestyle behaviors on the association between single-child status and HBP in boys (*n* = 25,661).
