# Supplementary material for: Exploring the Associations between Single-Child Status and Childhood High Blood Pressure and the Mediation Effect of Lifestyle Behaviors
Source: Nutrients. 2022 Jan 24;14(3):500. doi: 10.3390/nu14030500 (PMC8839388; doi:10.3390/nu14030500)
Supplement: Supplementary file 1 [file nutrients-14-00500-s001.zip › nutrients-1565962-supplementary.pdf]

Table S1. Moderation of lifestyle behaviors on the association between single-child status and BP z score in girls (*n*=25030).

Moderator	SBP			DBP		
	**Df**	**F value**	***P* value**	**Df**	**F value**	***P* value**
Meat consumption per week	3	1.053	0.368	3	0.953	0.414
Fruits consumption per week	3	1.093	0.350	3	0.772	0.509
Vegetable consumption per week	3	1.283	0.278	3	1.142	0.330
Beverage consumption per week	2	0.044	0.957	2	0.092	0.912
Physical activity	2	0.920	0.399	2	0.475	0.622
Sleeping duration	2	6.979	<0.001***	2	5.376	0.005**

Adjusted for age, birth weight, gestational age, breastfeeding, delivery mode, family history of hypertension, parental highest education degree, BMI z score. The moderation effect of sedentary behavior was examined by hierarchical multiple regression. The R-square variation was 0.000 and F statistics variation was 3.229 (*p*=0.072) for SBP and the R-square variation was 0.000 and F statistics variation was 0.169 (*p*=0.681) for DBP. ***p* <0.01， ****p* <0.001.

Table S2. Moderation of lifestyle behaviors on the association between single-child status and HBP in girls (*n*=25030).

Moderator	HBP	
	**OR (95%CI)**	***P* value**
Meat consumption per week		
0~1 day(s)	1 (ref)	
2~3 days	1.11 (0.86~1.43)	0.424
4~5 days	1.08 (0.82~1.41)	0.596
6~7 days	1.16 (0.91~1.48)	0.242
Fruits consumption per week		
0~1 day(s)	1 (ref)	
2~3 days	0.97 (0.68~1.40)	0.890
4~5 days	1.17 (0.83~1.67)	0.369
6~7 days	1.18 (0.84~1.66)	0.333
Vegetable consumption per week		
0~1 day(s)	1 (ref)	
2~3 days	0.79 (0.50~1.24)	0.305
4~5 days	1.12 (0.73~1.72)	0.599
6~7 days	1.11 (0.76~1.63)	0.575
Beverage consumption per week		
0 day	1 (ref)	
1~2 days	0.99 (0.97~1.02)	0.505
3 days and more	0.98 (0.95~1.02)	0.301
Physical activity		
Low intensity	1 (ref)	
Median intensity	1.00 (0.97~1.04)	0.985
High intensity	1.00 (0.97~1.04)	0.829
Sedentary behavior	1.00 (1.00~1.00)	0.601
Sleeping duration		
Adequate sleep	1 (ref)	
Insufficient sleep	1.03 (1.00~1.05)	0.042*
Hypersomnia	1.09 (1.00~1.19)	0.048*

Adjusted for age, birth weight, gestational age, breastfeeding, delivery mode, family history of hypertension, parental highest education degree, BMI z score. Abbreviation: HBP, high blood pressure. $^{*}p<0.05$.

Table S3. Mediation of lifestyle behaviors on the association between single-child status and BP in boys (n=25661).

Mediator	*n*	Outcome	Total association		Direct association		Indirect association		Mediation proportion
			β (95%CI)	*P*	β (95%CI)	*P*	β (95%CI)	*P*	
Meat consumption per week	20153	SBP z score	-0.033 (-0.065~-0.000)	0.047	-0.036 (-0.068~-0.004)	0.028	0.003 (0.001~0.006)	0.013^{**}	-10.72%
	20155	DBP z score	-0.014 (-0.046~0.018)	0.395	-0.017 (-0.049~0.015)	0.298	0.003 (0.001~0.006)	0.014^{**}	-22.37%
	20169	HBP	-0.068 (-0.140~0.004)	0.065	-0.071 (0.143~0.001)	0.054	0.003 (0.000~0.006)	0.025^{*}	-4.55%
Fruits consumption per week	20114	SBP z score	-0.034 (-0.066~-0.002)	0.039	-0.031 (-0.063~0.001)	0.060	—	—	—
	20116	DBP z score	-0.013 (-0.045~0.018)	0.410	-0.011 (-0.043~0.021)	0.489	—	—	—
	20130	HBP	-0.067 (-0.014~0.005)	0.069	-0.064 (-0.014~0.008)	0.082	—	—	—
Vegetable consumption per week	20157	SBP z score	-0.035 (-0.067~-0.003)	0.035	-0.035 (-0.067~-0.002)	0.035	—	—	—
	20159	DBP z score	-0.015 (-0.047~0.016)	0.341	-0.015(-0.047~0.016)	0.343	—	—	—
	20173	HBP	-0.071 (-0.143~0.001)	0.054	-0.071 (-0.143~0.002)	0.055	—	—	—
Beverage consumption per week	20038	SBP z score	-0.033 (-0.066~-0.001)	0.044	-0.034 (-0.066~-0.001)	0.041	0.001 (-0.000~0.001)	0.113	—
	20040	DBP z score	-0.014 (-0.045~0.018)	0.402	-0.014 (-0.046~0.018)	0.378	0.001 (-0.000~0.002)	0.088	—
	20054	HBP	-0.071 (-0.143~0.002)	0.056	-0.071 (0.143~0.002)	0.055	0.000 (-0.001~0.001)	0.748	—
Physical activity	18281	SBP z score	-0.022 (-0.056~0.012)	0.200	-0.022 (-0.056~0.012)	0.202	—	—	—
	18282	DBP z score	-0.000 (-0.034~0.033)	0.992	0.001 (-0.032~0.035)	0.939	—	—	—
	18295	HBP	-0.039 (-0.115~0.037)	0.312	-0.037 (-0.112~0.039)	0.345	—	—	—
Sedentary behavior	18368	SBP z score	-0.032 (-0.066~0.001)	0.060	-0.032 (-0.066~0.002)	0.061	—	—	—
	18368	DBP z score	-0.013 (-0.046~0.020)	0.444	-0.013 (-0.046~0.021)	0.453	—	—	—
	18381	HBP	-0.070 (-0.145~0.006)	0.071	-0.069 (-0.145~0.006)	0.072	—	—	—
Sleeping duration	19042	SBP z score	-0.034 (-0.068~-0.001)	0.042	-0.034 (-0.067~-0.001)	0.043	—	—	—

19044	DBP z score	-0.012 (-0.045~0.021)	0.472	-0.012 (-0.045~0.021)	0.477	—	—	—
19057	HBP	-0.070 (-0.144~0.005)	0.066	-0.070 (-0.001~0.000)	0.066	—	—	—

Note: The independent variable in model was single-child status and the reference group was "single children". Adjusted for age, birth weight, gestational age, breastfeeding, delivery mode, family history of hypertension, parental highest education degree, BMI z score. "—" represented the negative value of indirect association. Abbreviation: BP, blood pressure; SBP, systolic blood pressure; DBP, diastolic blood pressure; HBP, high blood pressure. **p*<0.05，***p* <0.01

Table S4. Moderation of lifestyle behaviors on the association between single-child status and BP z score in boys (*n*=25661).

Moderator	SBP			DBP		
	**Df**	**F value**	***P* value**	**Df**	**F value**	***P* value**
Meat consumption per week	3	0.961	0.410	3	0.923	0.429
Fruits consumption per week	3	1.249	0.290	3	0.787	0.501
Vegetable consumption per week	3	0.722	0.538	3	0.073	0.974
Beverage consumption per week	2	0.312	0.732	2	0.310	0.733
Physical activity	2	0.360	0.548	2	0.414	0.520
Sleeping duration	2	1.900	0.168	2	5.080	0.024*

Adjusted for age, birth weight, gestational age, breastfeeding, delivery mode, family history of hypertension, parental highest education degree, BMI z score. The moderation effect of sedentary behavior was examined by hierarchical multiple regression. The R-square variation was 0.000 and F statistics variation was 2.486 (*p*=0.115) for SBP and the R-square variation was 0.001 and F statistics variation was 1.042 (*p*=0.307) for DBP. **P*<0.05.

Table S5. Moderation of lifestyle behaviors on the association between single-child status and HBP in boys (*n*=25661).

Moderator	HBP	
	**OR (95%CI)**	***P* value**
Meat consumption per week		
0~1 day(s)	1 (ref)	
2~3 days	1.46 (0.79~1.39)	0.754
4~5 days	1.49 (0.79~1.40)	0.747
6~7 days	0.95 (0.73~1.23)	0.680
Fruits consumption per week		
0~1 day(s)	1 (ref)	
2~3 days	1.15 (0.87~1.52)	0.330
4~5 days	0.89 (0.67~1.17)	0.392
6~7 days	1.05 (0.81~1.36)	0.736
Vegetables consumption per week		
0~1 day(s)	1 (ref)	
2~3 days	0.99 (0.66~1.49)	0.957
4~5 days	1.30 (0.89~1.91)	0.182
6~7 days	1.14 (0.81~1.60)	0.458
Beverage consumption per week		
0 day	1 (ref)	

1~2 days	0.93 (0.78~1.09)	0.356
3 days and more	0.88 (0.73~1.06)	0.178
Physical activity		
Low intensity	1 (ref)	
Median intensity	0.83 (0.66~1.05)	0.117
High intensity	0.89 (0.72~1.10)	0.271
Sedentary behavior	0.98 (0.96~1.00)	0.035
Sleeping duration		
Adequate sleep	1 (ref)	
Insufficient sleep	1.19 (1.02~1.38)	0.024*
Hypersomnia	1.29 (0.83~1.99)	0.260

Adjusted for age, birth weight, gestational age, breast feeding, delivery mode, family history of hypertension, parental highest education degree, BMI z score. *p<0.05.
